# Evaluation of a Workplace Active Rest Program in Office Workers With Comparison of a Prospective and Retrospective Survey

**DOI:** 10.1177/00469580231220605

**Published:** 2023-12-25

**Authors:** Heidi Lehmann, Thomas Kraus, André Esser, Julia Krabbe

**Affiliations:** 1 Institute of Occupational, Social and Environmental Medicine, Medical Faculty, RWTH Aachen University, Aachen, Germany; 2 Social Accident Insurance Institution for the Energy, Textile, Electrical and Media Products Sectors (BG ETEM), Köln, Germany

**Keywords:** workplace active rest program, health promotion, health program, public administration, physical activity

## Abstract

Physical activity-related workplace interventions can be counterstrategies for physical inactivity due to office work. Newly introduced programs should be evaluated for success. This study aimed to evaluate the intervention of a workplace active rest program and to compare a prospective and retrospective design of evaluation. A Germany-wide multicenter evaluation of a 3-month workplace active rest program (30 min, once a week) was carried out at 14 locations with a longitudinal pre/post design by means of an anonymous questionnaire (n_pre_ = 405, n_post_ = 369). The participants’ program-related changes in targeted characteristics regarding posture, function, complaints and physical awareness were collected with a questionnaire in a prospective design and afterward retrospectively. The prospective evaluation showed a significant improvement in the target characteristic “postural muscles in the neck area”. In the retrospective survey, all target characteristics improved significantly. There were no differences between locations. The workplace active rest program in this study had positive effects on the perception of postural neck muscle status regardless of prospective or retrospective approach. Selection of survey mode should depend on desired outcome and consecutive influencing factors. In this specific case, retrospective survey could give more indirect information about overall satisfaction with the program and job although being influenced by response bias. Recall bias should be relatively small for shorter time periods assessed. Future studies should account for corresponding bias and specifics of target characteristics regardless of the chosen approach to survey.

**What do we already know about this topic?**
Physical activity during working time is decreasing considerably among work population while a sedentary lifestyle contributes to multiple risk factors and conditions regarding health.
**How does your research contribute to the field?**
Prospective and retrospective questionnaires were compared for evaluation of physical activity-related work program and important implications of approach of survey, as well as effects of physical activity-related interventions are discussed.
**What are your research’s implications towards theory, practice, or policy?**
A physical activity-related workplace active rest program can improve at least perception of postural neck musculature status and selection of survey mode should depend on desired outcome and consecutive influencing factors.

## Introduction and Objectives

Although it is widely known that a sedentary lifestyle is associated with serious consequences, comorbidities and economic burden^1-4^ working in a mainly sitting position is common for a growing number of adults.^5^ Interestingly, sedentary work seems to be associated with sedentary behavior outside of work^6^ aggravating effects of a sedentary lifestyle. Especially musculoskeletal symptoms (MSS), such as neck or lower back pain, are associated with lack of regular physical activity^7,8^ and reduced with increasing physical activity in office workers.^9-11^ The workplace represents a feasible opportunity to implement promotion for physical activity. Here, common barriers for preventing physical activity like lack of physical movement during work or lack of dedicated times for physical activities are present and could be addressed with targeted measures, for example, promotion of active commute to work, workplace counseling for physical activity or led activity sessions,^12^ especially due to the amount adults spend at work.^13^ Programs promoting physical activity at work are suitable for positively influencing work-related outcomes, for example, absenteeism, work performance and productivity.^14-16^ Additionally, positive effects on employees’ health include improvement in weight related outcomes and mental health.^17^

A typical physical activity-related measure is the workplace active rest, in which an activity program focuses on a short period of time and effort^18^ so that it can be implemented during work breaks. Reported positive effects include improvement in physical activity, mental health and personal relationships.^19,20^ A typical implementation of workplace active rest was reported by Michishita et al.^19^ In this study employees were instructed to participate in a workplace active rest program 3 times a week during lunch time for 10 weeks consisting of warm-up, cognitive functional training, aerobic exercise, resistance training and cool-down. After that, physical activity and job satisfaction had improved. In addition to assessment of physical activity and job satisfaction, evaluation of a physical activity-related measure can also target quality of life (QoL), musculoskeletal symptoms (MSS) or self-reported well-being.^21-24^

To ensure effectiveness of the chosen active rest program in the workplace an evaluation is recommended assessing consecutive targets. It can be conducted prospectively or in retrospect. Retrospective analysis is typically associated with less effort and less expensive which could be more attractive for employers and companies. Furthermore, retrospective assessment seems to be more associated with satisfaction with the result than prospective evaluation.^25^ Since satisfaction with a work-related physical activity measure is directly contribution to work engagement,^26^ retrospective evaluation could be favorable for workplace active rest programs. However, high cognitive abilities are required in subjects for retrospective surveys.^27^ With a retrospective period of no more than 3 months and with employees who are mentally healthy in the workplace context, the use of retrospective procedures seems justified.^28^

A workplace active rest was introduced in 2016 at an insurance institution in Germany. Its employees work at 14 locations throughout Germany and approximately 82% of the employees perform office work predominantly in a seated position. In the present study, employees who took part in the 3-month program were asked about posture, coordination, and body perception, as well as physical complaints regarding MSS. The survey was conducted before the measure (t0) and afterward (t1). At t1, the respondents were also asked about their memory of t0. This design was intended to test the following 2 hypotheses:

(1) The introduction of a 3-month workplace active rest has positive effects on posture, coordination, body perception and physical complaints regarding MSS,
(2) A purely retrospective survey is not inferior to a prospective survey regarding evaluation of health promotion measures.

## Methods

### Design and Timeline

**Figure 1.**
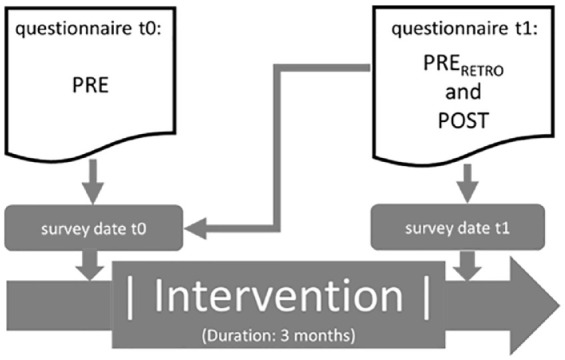
Timeline of intervention and survey dates.

This was a Germany-wide study on the evaluation of physical activity-related health promotion measures at 14 locations. For this purpose, pre- and post-status data were collected for various target characteristics (for a description of the questionnaires, see chapter “Surveys”). This was done both prospectively (using t0 and t1 questionnaires) and retrospectively (using t1 questionnaire), see Figure 1.

### Collective

All 1751 employees working at the insurance institution between 2016 and 2018 were contacted, informed about the intervention and had the opportunity to participate in the courses. Surveys were given out at the courses, so only participants could fill out the questionnaires. Out of the participants, 774 questionnaires, 405 at t0 and 369 at t1, were returned. Questionnaires were not marked or equipped with ID, so it was not possible to identify t0 and t1 of the same participants or determine the number of participants that answered both questionnaires. There was no control group without intervention that received the questionnaires.

Table 1 shows the grouped age of the responders and their gender. The categories go from “younger than 20 years” up to 60 years in equal steps. There were 404 valid cases for t0 with one questionnaire without age information excluded and 369 valid cases for t1. The median age in both surveys was the category 31 to 40 years. The relative frequencies in the age categories of both surveys were similar. 52.8% were female at t0 and 49.1% at t1. This was a non-representative sample since participation was voluntary and therefore factors that cannot be adjusted for could have influenced participation behavior, for example, satisfaction with the company, the program or survey.

**Table 1.** Frequency Count of Age Categories and Gender—t0 and t1 Surveys.

Survey time	t0 Questionnaire		t1 Questionnaire	
Age category	Frequency (n)	Percentages (%)	Frequency (n)	Percentages (%)
Younger than 20 years	71	17.6	67	18.2
21-30 years	84	20.8	77	20.9
31-40 years	82	20.3	67	18.2
41- 50 years	92	22.8	79	21.4
51-60 years	75	18.6	79	21.4
Total	404	100.0	369	100.0
	t0 questionnaire		t1 questionnaire	
Gender	Frequency (n)	Percentages (%)	Frequency (n)	Percentages (%)
Missing	1			
Male	191	47.2	188	50.9
Female	214	52.8	181	49.1
Total	405	100.0	369	100.0

### Interventions

The courses for workplace active rests were held weekly during working hours at 14 locations for a period of 3 months with a maximum of 12 participants per group. All trainers were trained in sports science according to the criteria of the statutory health insurance.^29^ Targeted objectives were strengthening of postural muscles, reduction of muscle tenseness, improvement of neck mobility, coordination and body perception and reduction in pain of neck, shoulders or back. The courses were all conducted similarly including warm up, exercises regarding mobility and flexibility, as well as muscle strengthening and endurance training overall for 30 min. Typical elements were ball games, treadmill training, lying exercises for abdominal and back muscle strengthening and standing exercises training coordination and flexibility.

### Surveys

Surveys were primarily used and communicated as in-house evaluation of the workplace active rest program. Scientific assessment was planned and conducted afterward although participants were informed during the survey that this could follow. Questionnaire items were adapted from a general habitual well-being questionnaire being popular in Germany and orientated on the aimed objectives and positive changes in target characteristics. The development of the questionnaire was based on the validated questionnaire on general habitual well-being by Georg Wydra (original instrument in German).^30^ Item 5.1 was inspired by the “general questionnaire of Vita 40+”^31^(p. 40), and item 7.1 was inspired by the essay by Bös and Wydra.^32^

The questionnaire is included in the Supplemental Material in English translation (Supplemental Material questionnaire t0 and t1).

Target characteristics included items regarding self-report of status of postural musculature, muscle tension, neck mobility, coordination, body awareness, stress, feeling well and balanced, as well as complaints regarding different muscle groups, exercise in leisure time and during breaks, fitness and knowledge regarding back pain.

See the following example items:

• My postural muscles in the neck area are very good.
• I very rarely have tension.
• I very rarely have complaints in the neck area.

For all items see Supplemental questionnaires t0 and t1.

A 7-point scale was chosen with regard to sensitivity to change. The response alternatives are: 1 = not true at all, 2 = mostly not true, 3 = somewhat untrue, 4 = moderately true, 5 = somewhat true, 6 = mostly true 7 = almost completely true. This questionnaire was named “Posture, Function, Complaints and Physical Awareness” (PFCPA). Furthermore, the following item was added “I have stress at work o no, o little, o moderate, o a lot of.” The items for the survey of status were constructed of a 7 scale the Likert-type with a view to a high sensitivity to change.

All employees from 2016 to 2018 were interviewed using paper-pencil or electronically during working hours at the respective location. The survey was voluntary and anonymous, so participants of the interventions could choose to not participate in the survey. As the working language is German, a German questionnaire version was used.

Participants received a t0 questionnaire from their trainer at the beginning of the first meeting. Shortly before the end of the 3-month measure, the participants received a t1 questionnaire from their trainers. All respondents were given the opportunity to ask questions. After completion, the questionnaires were first put into an envelope to ensure anonymity and then thrown into an urn.

**Approval of the research protocol:** The study was conducted in accordance with the Declaration of Helsinki and approved by the Ethics Committee of the Medical Faculty, RWTH Aachen University (EK 22-437).

**Informed Consent:** Participation was voluntary. Participants received the in the questionnaire included information with the option to inquiry further and could decide to answer the questionnaire or not.

### Statistical Analysis

Since the evaluation was initially planned as in-house evaluation of the active rest program, statistical approach beyond descriptive analysis was not planned beforehand. Accordingly, no sample size estimation was conducted beforehand. In retrospect the statistical analysis chosen had to account for the specifics of study design: Since questionnaires in the prospective design could not be matched for t0 and t1 and it cannot be guaranteed that both groups included the same participants an analysis for independent samples had to be used. For retrospective analysis the matching of both time points, post (t1) and pre_retro_ (t1), was possible so an analysis for dependent samples was chosen. Accordingly, the retrospective evaluation was carried out using repeated measures analysis of covariance (ANCOVA), controlling for age, gender, activity at work, stress, self-reported health status, and location. The prospective analysis carried out using of the Mann-Whitney-*U* test. The comparison of the pre-status (t0) with the pre_retro_ (t1) is also carried out using the Mann-Whitney-*U* test. Data analysis was performed with. SPSS Statistics for Windows, version 27.0 (IBM Corp. Armonk, NY). First, descriptive data analyses were carried out, in which the statistical parameters mean, standard deviations, median and range were determined. Connected samples were analyzed by means of SPSS repeated measures analyses of variance with covariates using the general linear model (GLM) and unconnected samples were analyzed with the Mann-Whitney U test. Significance levels were assumed to be *P* < .05 as significant, *P* < .01 as highly significant. The graphical representation of the results was done using Microsoft Excel for Microsoft 365 MSO (Microsoft Corporation, Redmond, WA).

## Results

It was examined to what extent the subjective classification of the target characteristics improved during the measure. The following results refer to the evaluation of the t1 questionnaire as a retrospective evaluation. This involves the comparison of the post (t1) with the pre_retro_ (t1) with adjustments for age, gender, stress, state of health and exercise at work as well as taking into consideration the location—see Table 2. Post and pre_retro_ refer to the same persons in each case, so they are related samples. The respective target characteristics were not served and recorded by the measure at all locations, but only where they matched the course content (variable n per target characteristic).

**Table 2.** ANCOVA of Post (t1) and Pre_retro_ (t1) by Location, Controlled for Age, Sex, Stress, Exercise at Work and Health Status.

Target characteristic	N	Intra-subject effects			location effect		
		*F*	df	Eta²	*F*	df	Eta²
Postural muscles in the neck area	195	12.919***	1	0.068	0.283	12	0.019
Postural muscles in the shoulder area	194	25.925***	1	0.128	0.514	12	0.034
Postural muscles in the back area	195	35.144***	1	0.166	0.598	12	0.039
Postural muscles in the abdomen area	139	3.829	1	0.030	0.428	8	0.027
Complaints neck area	203	17.050***	1	0.085	1.074	13	0.071
Complaints shoulder area	202	24.240***	1	0.117	1.238	13	0.081
Complaints back area	202	39.405***	1	0.177	0.959	13	0.064
Mobility neck area	193	14.700***	1	0.077	1.151	11	0.067
Complaints lumbar region (lower back)	187	27.480***	1	0.140	1.233	12	0.081
Tension	196	28.516***	1	0.138	0.837	12	0.053
Coordination	40	3.244	1	0.090	0.017	1	0.001
Body awareness	56	0.152	1	0.003	0.775	2	0.031

*** *P* ≤ .001.

The comparison of the post (t1) with the pre_retro_ (t1) shows significant results in line with expectations for the majority of the intra-subject effects. With regard to the intra-subject effects, there were significant differences with regard to the following target characteristics: Neck postural muscles, shoulder postural muscles, back postural muscles, neck mobility, neck complaints, shoulder complaints, back complaints and lower back complaints. There were no significant location effects for any of the target characteristics.

The results of the prospective evaluation are as follows: the analysis in Table 3 compares the pre collected at time t0 with the post collected at time t1. The Mann-Whitney-*U* test for this shows the following results—see Table 3.

**Table 3.** Mann-Whitney-U Test—Comparison of Pre (t0) and Post (t1).

Target characteristic	N		*U*	SE
	Pre	Post		
Postural muscles in the neck area	388	195	41 630.0*	1877.457
Postural muscles in the shoulder area	387	195	36 551.0	1869.533
Postural muscles in the back area	387	195	39 815.5	1871.176
Postural muscles in the abdomen area	294	141	20 336.5	1196.957
Complaints neck area	404	203	40 676.0	1989.301
Complaints shoulder area	405	203	40 159.0	1995.366
Complaints back area	404	202	38 096.0	1983.271
Mobility neck area	378	193	34 203.0	1820.431
Complaints lumbar region (lower back)	371	187	32 934.0	1755.300
Tension	387	196	36 485.0	1875.449
Coordination	62	40	1489.5	142.353
Body awareness	104	56	3118.5	276.451

* *P* ≤ .05.

Table 3 shows a significant difference between the groups for the characteristic “postural muscles neck area” in the conventional pre-post comparison using the Mann-Whitney *U*-test.

In view of the differences found between retrospective comparison (post (t1) vs pre_retro_ (t1)) and prospective comparison (conventional pre-post comparison, ie, post (t1) vs pre (t0)), we were interested in whether both pre ((t0) and (t1)) differ. The following table shows this using the Mann-Whitney-*U* test—see Table 4.

**Table 4.** Mann-Whitney-*U* test Comparison of measured Pre (t0) and Pre_retro_ (t1).

Target characteristic	N		*U*	SE
	Pre (t0)	Pre (t1)		
Postural muscles in the neck area	388	195	17 810.0 ***	1886.890
Postural muscles in the shoulder area	387	194	14 566.5 ***	1876.740
Postural muscles in the back area	387	195	14 000.0 ***	1881.362
Postural muscles in the abdomen area	294	139	7019.0 ***	1196.375
Complaints neck area	404	203	17 189.0 ***	2009.269
Complaints shoulder area	405	202	17 380.5 ***	2008.542
Complaints back area	404	203	15 969.0 ***	2011.132
Mobility neck area	378	193	15 980.0 ***	1838.119
Complaints lumbar region (lower back)	371	188	13 283.0 ***	1779.747
Tension	387	196	16 039.5 ***	1895.577
Coordination	62	40	545.5***	144.054
Body awareness	104	56	1821.5***	275.565

*** *P* ≤ .001.

**Figure 2.**
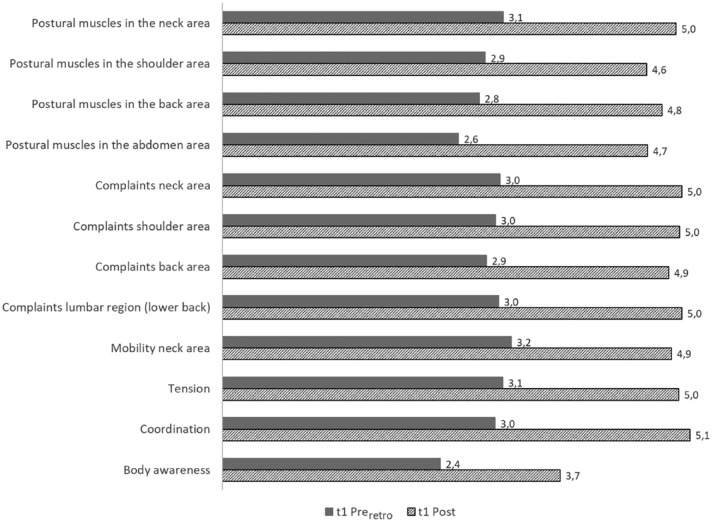
Mean comparison t1-survey Post - Pre_retro._

The Mann-Whitney-*U* test showed a significant difference between the pre (t0) and the pre_retro_ (t1) for all examined characteristics for the 5% significance level (also see Figure 2). For all these characteristics, it made a significant difference whether the pre was measured at the time (t0) or retrospectively (t1). For the pre (t0), a more positive state was indicated than for the pre_retro_ (t1).

## Discussion

In our study, employees were asked to evaluate their posture, coordination, and body perception as well as physical complaints in questionnaire prospectively and retrospectively after having taken part in a workplace active rest program. The prospective evaluation showed a significant change in the target characteristic “postural muscles in the neck area”. For all target characteristics, there were no differences between locations. In the retrospective survey, all target characteristics appeared to have changed significantly.

Workplace active rest, a short activity program which can be implemented during work breaks, is a physical activity related workplace measure with reported effects including improvement in physical activity, mental health and personal relationships. Aside assessment of physical activity, job satisfaction quality of life (QoL), and MSS can also be evaluated.^19-24^ Interestingly, MSS are associated with psychosocial factors such as job demands and control, social support and job satisfaction.^33^ So, targeting MSS with corresponding workplace measures could also bring benefits regarding job satisfaction. In this study the retrospective survey identified self-reported improvements of postural musculature, muscle tension and neck mobility, but not regarding coordination and body awareness. Accordingly, studies assessing the effects of workplace physical activity-related measures also reported improvement regarding MSS and especially pain.^34^ Caputo et al. introduced a 7 weeks long intervention with supervised exercises including resistance exercises or stretching and postural exercises.^10,24,34,35^ After that, pain decreased in both groups while health related QoL was unchanged. In a considerably longer 9 months long intervention with a similar exercise structure compared to the workplace active rest in this study in metal workers, a considerable reduction in neck pain was reported.^10^ Another study also reported reductions in self-reported pain in postural musculature after a 12 weeks long intervention in office workers.^24^ Similar findings were reported for similar interventions in office workers^36-38^ suggesting that interventions in the workplace are feasible to improve perceived pain in postural musculature in mainly sitting workers even with interventions with training only once a week and for short periods such as 3 months. Interestingly, those studies reported both perceived improvements for example, in pain scales^36,37^ and objective improvements for example, in postural angles^37^ or days of sick leave.^38^ Furthermore, not only pain but also psychosocial dimensions as QoL^36,39^ and job satisfaction^19,40^ were affected. Especially job satisfaction seems to be positively influenced by physical activity^41^ and physical activity-related interventions.^40^ However, physical activity in leisure time seems to be associated with a higher job satisfaction^42-44^ and a lower job satisfaction with the development of neck pain.^45^ Thus, the increase in physical activity per se and not necessarily during work time seems to exert the reported effects.

So, in view of the current state of the literature it was not surprising that even the rather short intervention in this study with a workplace active rest program for 30 min once a week demonstrated perceived improvements in postural musculature and pain complaints. Most target characteristics improved in the assessment of positive statements about these characteristics from somewhat untrue to higher agreement values, such as somewhat true, so a generally positive perception of those. This was true for all locations and no differences were detectable. Thus, although slight differences in exercises and different trainers were part of the intervention at different locations those circumstances seemed to have no major influences on the target characteristics, probably due to the definition of common goals and objectives. It has to be taken into consideration that the reported improvements are not directly related to an increase in physical activity or the intervention itself. The mere expectation from the team implementing the program could have influenced the perceived success of it, also known as Pygmalion effect.^46^ Since this was a longitudinal observation without control group, even coincidental findings cannot be excluded. However, an increase in job satisfaction and/or overall well-being would be even beneficial for employees and employers if it was not due to the intervention and an increase in physical activity. Since the evaluation was initially planned as in-house evaluation of the active rest program, statistical approach beyond descriptive analysis was not planned beforehand. Accordingly, no sample size estimation was conducted beforehand. This approach is a clear limitation of the study. However, we think that the findings of this study are valuable nevertheless since they were acquired from a real-life situation.

The registered effects in this study were mainly detectable for retrospective analysis. In prospective design only a perceivable improvement in neck musculature was reported. The prospective and retrospective classifications thus seem to assess the same characteristic significantly differently. Tashjian et al report that retrospective assessment seems to be more associated with satisfaction with the result than prospective evaluation.^25^ However, others report reports of greater effects and stress the danger of overestimating effects in retrospect.^47-49^ Furthermore, retrospective analysis seems to be influenced by overall experience with the process.^50^ If report of specific events or frequency is required, prospective records seem to be more accurate.^49^ Especially records of socially dismissed behavior or symptoms like heavy drinking^51^ or depression^52^ seem to be more accurate when collected prospectively and not as a retrospective report. Consecutively, recall bias is always a potential source of distortion of received results in retrospective surveys.^53^ Furthermore, high cognitive abilities are required in subjects for retrospective surveys.^27^ However, healthy participants should be able to reliably recall up to 3 months.^28^ Prospective surveys can suffer from response shift bias, indicating that the participants’ understanding of the response scale shifted during the study due to change of internal standards of interpretation in terms of scale calibration.^53^ Response shift has been reported in association with MSS^54^ or surgery^55,56^ although resulting in rather small effects.^54,56^ This could be a realistic explanation for the lack of detected improvements in the prospective setting as the standards regarding muscle strength or posture could have changed with the intervention. However, due to the study design possible effects could not be attributed to response shift certainly.

Another bias worth considering regarding this study is response bias. Generally, assessment of physical activity is both reported to be affected^57^ and not to be affected by response bias.^58^ Regarding physical activity social desirability as tendency of subjects to respond consistently with social norms could influence answers and even willingness to participate the survey. Over-reporting of physical activity seems to be common.^59,60^ Response bias seems to be based on both individual characteristics like gender or age,^61^ and existence and level of MSS.^62^ Workers with pain seem to response with a higher probability than without symptoms leading to overestimation of prevalence in studies with lower response rates.^62^ Pain report seems to be less influenced by social desirability than depressive or anxiety symptoms.^63^ So, for this study it is likely that participants with lower levels of pain in postural musculature and participants that experienced less effects would have participated less likely in the survey. Accordingly, a lower agreement with good postural musculature status is reported by the participants. Due to the lack of control group, the response bias could not be quantified. Due to the voluntary participation in both the courses and the survey, a pre-selected group was present. It can be assumed that people who took part in the health courses had a generally positive attitude toward physical activity and hoped that physical activity would have positive effects on the target characteristics surveyed in the study being also more affected by response shift in their answers. Additionally, the popular belief that exercise has positive effects on health and especially muscle and back related pain could have shaped the perception of improvements in subjects.^64,65^

## Conclusion

The workplace active rest program assessed as intervention ins this study with 30 min once a week over 12 weeks had positive effects on the perception of postural neck muscle status regardless of prospective or retrospective approach.

Prospective and retrospective survey regarding physical activity, MSS and pain seem to be both reasonable. Selection of survey mode should depend on desired outcome and consecutive influencing factors. Related bias should be accustomed for accordingly. In this specific case, retrospective survey could give more indirect information about overall satisfaction with the program and job although being influenced by response bias. Recall bias should be relatively small for shorter time periods assessed.

Future studies should account for corresponding bias and specifics of target characteristics regardless of the chosen approach to survey.

## Supplemental Material

sj-docx-1-inq-10.1177_00469580231220605 – Supplemental material for Evaluation of a Workplace Active Rest Program in Office Workers With Comparison of a Prospective and Retrospective Survey Click here for additional data file. Supplemental material, sj-docx-1-inq-10.1177_00469580231220605 for Evaluation of a Workplace Active Rest Program in Office Workers With Comparison of a Prospective and Retrospective Survey by Heidi Lehmann, Thomas Kraus, André Esser and Julia Krabbe in INQUIRY: The Journal of Health Care Organization, Provision, and Financing
