# Supplementary material for: Evaluation of a Workplace Active Rest Program in Office Workers With Comparison of a Prospective and Retrospective Survey
Source: Inquiry. 2023 Dec 25;60:00469580231220605. doi: 10.1177/00469580231220605 (PMC10752044; doi:10.1177/00469580231220605)
Supplement: sj-docx-1-inq-10.1177_00469580231220605 – Supplemental material for Evaluation of a Workplace Active Rest Program in Office Workers With Comparison of a Prospective and Retrospective Survey [file sj-docx-1-inq-10.1177_00469580231220605.docx]

***Suppl. Material questionnaire t0 and t1***

**Suppl. Questionnaire t0**

**T0: Management Safety and Health (MSH) – feedback form „workplace active rest“**

Dear Ladies and Gentlemen,

We would like to tailor offers in the workplace health promotion to your needs. Therefore, we would like to ask you to answer this questionnaire today - i.e., before the actual start of the course. In a few weeks towards the end of the course, we will ask you the same questions again. Please complete the questionnaire and return it to the collection box. The questions are about your general well-being. The survey is completely anonymous. The data will be analysed by the MSH and, in addition to scientific purposes, will be used to improve the services offered.

Please do not hesitate to contact me if you have any further questions.

Thank you for your effort and stay healthy!

**Start of the questionnaire:**

1. **Age:** □ younger than 20 years, □ 21-30, □ 31-40; □ 41-50, □ 51-60, □ 61-70
2. **Gender:** □ male; □ female
3. **C. Are you below or above your normal weight, which answer applies to you?** (Normal weight = height in centimetres minus 100, e.g. 178cm=78 KG normal weight)

□ more than 10 kilos above my normal weight, □ 6-10 kilos above, □ 3-5 kilos above,

□ approximately normal weight, □ 3-5 below, □ more than 5 Kilo below

1. I have activity at work: □ not; □ little; □ medium; □ much
2. I have stress at work: □ not; □ little; □ medium; □ much
3. How do you rate your current state of health compared to others of your age and gender?

□ considerably worse,
□ worse,
□ slightly worse,
□ exactly the same,
□ slightly better,
□ better,
□ considerably better.

1. Do you have any other comments, ideas or requests for MSG?

Continued on the other side

**How are you currently, that is before you have attended classes?**

| **Nr.** | *Please only one cross in each line* **¡** | **Does not apply at all** | **Does mostly not apply** | **Applies little** | **Applies somewhat** | **Applies a little** | **Does mostly apply** | **Does almost completely apply** |
| --- | --- | --- | --- | --- | --- | --- | --- | --- |
| 1.1 | My postural musculature in the neck area is very good. | ➀ | ➁ | ➂ | ➃ | ➄ | ➅ | ➆ |
| 1.2 | My postural musculature in the shoulder area is very good. | ➀ | ➁ | ➂ | ➃ | ➄ | ➅ | ➆ |
| 1.3 | My postural musculature in the back area is very good. | ➀ | ➁ | ➂ | ➃ | ➄ | ➅ | ➆ |
| 1.4 | My postural musculature in the abdomen area is very good. | ➀ | ➁ | ➂ | ➃ | ➄ | ➅ | ➆ |
| 2.1 | I very rarely have tension. | ➀ | ➁ | ➂ | ➃ | ➄ | ➅ | ➆ |
| 3.1 | My mobility in the neck area is very good. | ➀ | ➁ | ➂ | ➃ | ➄ | ➅ | ➆ |
| 4.1 | My coordination is very good. | ➀ | ➁ | ➂ | ➃ | ➄ | ➅ | ➆ |
| 5.1 | My body awareness is very good. | ➀ | ➁ | ➂ | ➃ | ➄ | ➅ | ➆ |
| 6.1 | I rarely have stress. | ➀ | ➁ | ➂ | ➃ | ➄ | ➅ | ➆ |
| 7.1 | I almost always feel very well. | ➀ | ➁ | ➂ | ➃ | ➄ | ➅ | ➆ |
| 7.2 | I almost always feel very balanced. | ➀ | ➁ | ➂ | ➃ | ➄ | ➅ | ➆ |
| 8.1 | I very rarely have complaints in the neck area. | ➀ | ➁ | ➂ | ➃ | ➄ | ➅ | ➆ |
| 8.2 | I very rarely have complaints in the shoulder area. | ➀ | ➁ | ➂ | ➃ | ➄ | ➅ | ➆ |
| 8.3 | I very rarely have complaints in the back area. | ➀ | ➁ | ➂ | ➃ | ➄ | ➅ | ➆ |
| 8.4 | I very rarely have complaints in the lumbar region (lower back). | ➀ | ➁ | ➂ | ➃ | ➄ | ➅ | ➆ |
| 9.1 | I exercise very often in my free time. | ➀ | ➁ | ➂ | ➃ | ➄ | ➅ | ➆ |
| 9.2 | I move very often during breaks. | ➀ | ➁ | ➂ | ➃ | ➄ | ➅ | ➆ |
| 10.1 | I have some colleagues whom I can really trust. | ➀ | ➁ | ➂ | ➃ | ➄ | ➅ | ➆ |
| 11.1 | I have important knowledge about the development of back pain. | ➀ | ➁ | ➂ | ➃ | ➄ | ➅ | ➆ |
| 12.1 | I am very fit as compared to my comrades in age and gender. | ➀ | ➁ | ➂ | ➃ | ➄ | ➅ | ➆ |
| 12.2 | My physical performance during work is very good. | ➀ | ➁ | ➂ | ➃ | ➄ | ➅ | ➆ |

**Thank you for your effort.**

**Suppl. Questionnaire t1**

**T1: Management Safety and Health (MSH) – feedback form „workplace active rest“**

Dear Ladies and Gentlemen,

We would like to tailor offers in the workplace health promotion to your needs. In order for us to succeed, please answer the following questions and put the three-page questionnaire into the collection box. The survey is completely anonymous. The data will be analysed by the MSH and, in addition to scientific purposes, will be used to improve our services.

Please do not hesitate to contact me if you have any further questions.

Thank you for your effort and stay healthy!

Start of the questionnaire:

1. **Age:** **:** □ younger than 20 years, □ 21-30, □ 31-40; □ 41-50, □ 51-60 □ 61-70
2. **Gender:** □ male □ female
3. **I have activity at work**: □ not; □ little; □ medium; □ much
4. **I have stress at work:** □ not; □ little; □ medium; □ much
5. How do you rate your current state of health compared to others of your age and gender?

□ considerably worse, □ worse, □ slightly worse, □ exactly the same,

□ slightly better, □ better, □ considerably better.

1. F. Have you participated in the workplace active rest program □ yes.

□ No because….. □ I did not find the offer appealing.

□ I did not have time/was out of the house.

□ Other reasons: ________________________________________________________

**If you did not take part in the moving break, the survey ends here. Please return the questionnaire to the collection box. Thank you for your cooperation,**

1. What did you like best about the workplace active rest programme?

________________________________________________________________________________________

1. What did you not like? What can we still improve?

________________________________________________________________________________________

1. Do you have any other comments, praise, criticism, ideas or suggestions??

________________________________________________________________________________________

1. What is the impact of the workplace active rest programme on you? Please rate the following statements on pages 2 and 3.

|  | How are you **currently** after participation in the programme?  How did you feel **previously**, i.e. before you had started the course  *Please put only* ***one*** *cross in each line* **¡** | **Does not apply at all** | **Does mostly not apply** | **Applies little** | **Applies somewhat** | **Applies a little** | **Does mostly apply** | **Does almost completely apply** |
| --- | --- | --- | --- | --- | --- | --- | --- | --- |
| **1.1** | **Currently:** My postural musculature in the neck area is very good. | ➀ | ➁ | ➂ | ➃ | ➄ | ➅ | ➆ |
|  | **Previously**: My postural musculature in the neck area is very good. | ➀ | ➁ | ➂ | ➃ | ➄ | ➅ | ➆ |
| **1.2** | **Currently**: My postural musculature in the shoulder area is very good. | ➀ | ➁ | ➂ | ➃ | ➄ | ➅ | ➆ |
|  | **Previously**: My postural musculature in the shoulder area is very good. | ➀ | ➁ | ➂ | ➃ | ➄ | ➅ | ➆ |
| **1.3** | **Currently**: My postural musculature in the back area is very good. | ➀ | ➁ | ➂ | ➃ | ➄ | ➅ | ➆ |
|  | **Previously**: My postural musculature in the back area is very good. | ➀ | ➁ | ➂ | ➃ | ➄ | ➅ | ➆ |
| **1.4** | **Currently**: My postural musculature in the abdomen area is very good. | ➀ | ➁ | ➂ | ➃ | ➄ | ➅ | ➆ |
|  | **Previously**: My postural musculature in the abdomen area is very good. | ➀ | ➁ | ➂ | ➃ | ➄ | ➅ | ➆ |
| **2.1** | **Currently**: I very rarely have tension. | ➀ | ➁ | ➂ | ➃ | ➄ | ➅ | ➆ |
|  | **Previously**: I very rarely have tension. | ➀ | ➁ | ➂ | ➃ | ➄ | ➅ | ➆ |
| **3.1** | **Currently**: My mobility in the neck area is very good. | ➀ | ➁ | ➂ | ➃ | ➄ | ➅ | ➆ |
|  | **Previously**: My mobility in the neck area is very good. | ➀ | ➁ | ➂ | ➃ | ➄ | ➅ | ➆ |
| **4.1** | **Currently**: : My coordination is very good. | ➀ | ➁ | ➂ | ➃ | ➄ | ➅ | ➆ |
|  | **Previously**: My coordination is very good. | ➀ | ➁ | ➂ | ➃ | ➄ | ➅ | ➆ |
| **5.1** | **Currently**: My body awareness is very good. | ➀ | ➁ | ➂ | ➃ | ➄ | ➅ | ➆ |
|  | **Previously**: My body awareness is very good. | ➀ | ➁ | ➂ | ➃ | ➄ | ➅ | ➆ |
| **6.1** | **Currently**: I rarely have stress. | ➀ | ➁ | ➂ | ➃ | ➄ | ➅ | ➆ |
|  | **Previously**: I rarely have stress. | ➀ | ➁ | ➂ | ➃ | ➄ | ➅ | ➆ |
| **7.1** | **Currently**: I almost always feel very well. | ➀ | ➁ | ➂ | ➃ | ➄ | ➅ | ➆ |
|  | **Previously**: I almost always feel very well. | ➀ | ➁ | ➂ | ➃ | ➄ | ➅ | ➆ |
| **7.2** | **Currently**: I almost always feel very balanced. | ➀ | ➁ | ➂ | ➃ | ➄ | ➅ | ➆ |
|  | **Previously**: I almost always feel very balanced. | ➀ | ➁ | ➂ | ➃ | ➄ | ➅ | ➆ |

|  | How are you **currently**, i.e. after you have participated for a few hours / after having participated for several hours.  How were you **previously**, i.e. before you had started with the course?  *Please put only one cross in each line* **¡** | **Does not apply at all** | **Does mostly not apply** | **Applies little** | **Applies somewhat** | **Applies a little** | **Does mostly apply** | **Does almost completely apply** |
| --- | --- | --- | --- | --- | --- | --- | --- | --- |
| **8.1** | **Currently:** I very rarely have complaints in the neck area. | ➀ | ➁ | ➂ | ➃ | ➄ | ➅ | ➆ |
|  | **Previously:** I very rarely have complaints in the neck area. | ➀ | ➁ | ➂ | ➃ | ➄ | ➅ | ➆ |
| **8.2** | **Currently:** I very rarely have complaints in the shoulder area. | ➀ | ➁ | ➂ | ➃ | ➄ | ➅ | ➆ |
|  | **Previously:** I very rarely have complaints in the shoulder area. | ➀ | ➁ | ➂ | ➃ | ➄ | ➅ | ➆ |
| **8.3** | **Currently:** I very rarely have complaints in the back area. | ➀ | ➁ | ➂ | ➃ | ➄ | ➅ | ➆ |
|  | **Previously:** I very rarely have complaints in the back area. | ➀ | ➁ | ➂ | ➃ | ➄ | ➅ | ➆ |
| **8.4** | **Currently:** I very rarely have complaints in the lumbar region (lower back). | ➀ | ➁ | ➂ | ➃ | ➄ | ➅ | ➆ |
|  | **Previously:** I very rarely have complaints in the lumbar region (lower back)**.** | ➀ | ➁ | ➂ | ➃ | ➄ | ➅ | ➆ |
| **9.1** | **Currently:** I exercise very often in my free time. | ➀ | ➁ | ➂ | ➃ | ➄ | ➅ | ➆ |
|  | **Previously:** I exercise very often in my free time. | ➀ | ➁ | ➂ | ➃ | ➄ | ➅ | ➆ |
| **9.2** | **Currently:** I move very often during breaks. | ➀ | ➁ | ➂ | ➃ | ➄ | ➅ | ➆ |
|  | **Previously:** I move very often during breaks. | ➀ | ➁ | ➂ | ➃ | ➄ | ➅ | ➆ |
| **10.1** | **Currently:** I have some colleagues whom I can really trust. | ➀ | ➁ | ➂ | ➃ | ➄ | ➅ | ➆ |
|  | **Previously:** I have some colleagues whom I can really trust. | ➀ | ➁ | ➂ | ➃ | ➄ | ➅ | ➆ |
| **11.1** | **Currently:** I have important knowledge about/ on the development of back pain. | ➀ | ➁ | ➂ | ➃ | ➄ | ➅ | ➆ |
|  | **Previously:** I have important knowledge about/ on the development of back pain. | ➀ | ➁ | ➂ | ➃ | ➄ | ➅ | ➆ |
| **12.1** | **Currently:** I am very fit as compared to my comrades in age and gender. | ➀ | ➁ | ➂ | ➃ | ➄ | ➅ | ➆ |
|  | **Previously:** I am very fit as compared to my comrades in age and gender. | ➀ | ➁ | ➂ | ➃ | ➄ | ➅ | ➆ |
| **12.2** | **Currently:** My physical performance during work is very good. | ➀ | ➁ | ➂ | ➃ | ➄ | ➅ | ➆ |
|  | **Previously:** My physical performance during work is very good. | ➀ | ➁ | ➂ | ➃ | ➄ | ➅ | ➆ |
